# Non-Canonical Thinking for Targeting ALK-Fusion Onco-Proteins in Lung Cancer

**DOI:** 10.3390/cancers9120164

**Published:** 2017-11-30

**Authors:** Wei Wu, Franziska Haderk, Trever G. Bivona

**Affiliations:** 1 Helen Diller Family Comprehensive Cancer Center, San Francisco, CA 94115, USA; wei.wu@ucsf.edu (W.W.); Franziska.Haderk@ucsf.edu (F.H.); 2 Department of Medicine, University of California, San Francisco, CA 94115, USA

**Keywords:** anaplastic lymphoma kinase, ALK, dimerization inhibition, neoantigens, CAR-T, immunotherapy

## Abstract

Anaplastic lymphoma kinase (*ALK*) gene rearrangements have been identified in lung cancer at 3–7% frequency, thus representing an important subset of genetic lesions that drive oncogenesis in this disease. Despite the availability of multiple FDA-approved small molecule inhibitors targeting ALK fusion proteins, drug resistance to ALK kinase inhibitors is a common problem in clinic. Thus, there is an unmet need to deepen the current understanding of genomic characteristics of ALK rearrangements and to develop novel therapeutic strategies that can overcome ALK inhibitor resistance. In this review, we present the genomic landscape of ALK fusions in the context of co-occurring mutations with other cancer-related genes, pointing to the central role of genetic epistasis (gene-gene interactions) in ALK-driven advanced-stage lung cancer. We discuss the possibility of targeting druggable domains within ALK fusion partners in addition to available strategies inhibiting the ALK kinase domain directly. Finally, we examine the potential of targeting ALK fusion-specific neoantigens in combination with other treatments, a strategy that could open a new avenue for the improved treatment of ALK positive lung cancer patients.

## 1. Introduction

**Figure 1:**
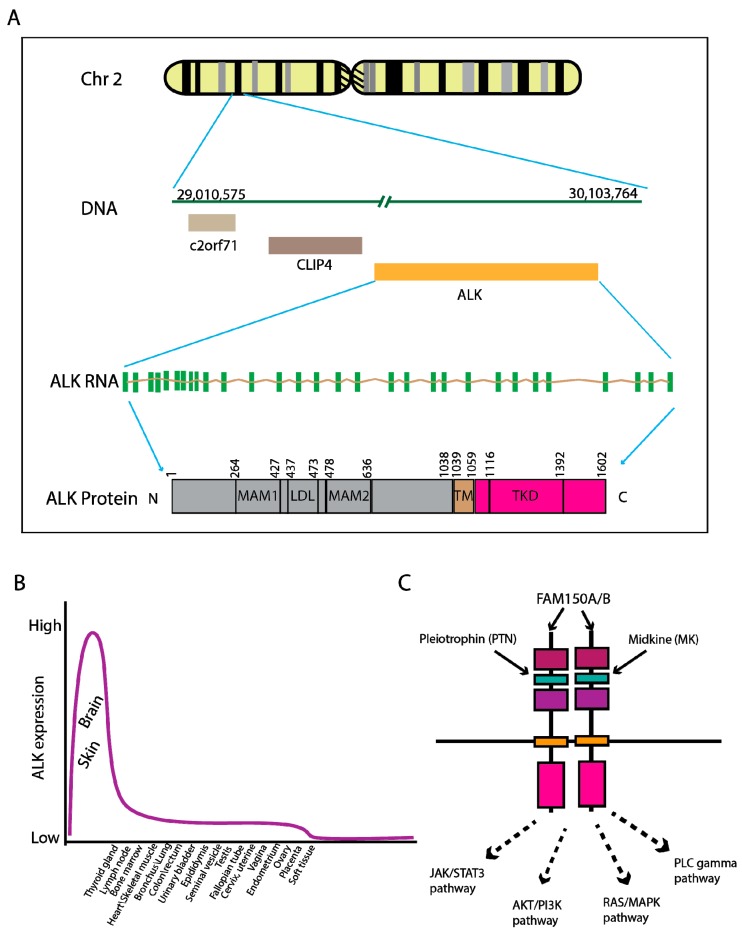
Genomic location and expression of *ALK* in physiological condition. (**A**) The human *ALK* gene is located on Chr2 p23.2–23.1 and expresses an RNA transcript of approximately 6267 kb encompassing 29 exons. The encoded protein contains 1602 amino acids with extracellular domains of two meprin, A-5 protein, multiple receptor protein-tyrosine phosphatase mu (MAM) regions and a low-density lipoprotein receptor domain class A (LDLa), a transmembrane domain (TM) and intracellular kinase domain (TKD). (**B**) The distribution of normal ALK protein expression in a variety of tissues/organs. adopted from the human protein atlas [2]. (**C**) ALK is a receptor tyrosine kinase. It is activated by ligand-binding and subsequent phosphorylation, transduces signaling through well-characterized JAK/STAT3, PI3K/AKT, RAS/MAPK, and PLC-gamma pathways. Three known ligands are: pleiotrophin (PTN), Midkin (MK) and FAM150A/B.

The human Anaplastic lymphoma kinase (*ALK*) gene is located on chromosome 2p23.2 and encodes an enzymatic protein, also known as ALK tyrosine kinase receptor or CD246 (cluster of differentiation 246). The ALK receptor tyrosine kinase belongs to the insulin receptor superfamily. This protein comprises an extracellular domain, a hydrophobic stretch corresponding to a single pass transmembrane region, and an intracellular kinase domain [1] (Figure 1A). The expression levels of ALK mRNA and protein are diminished after birth and maintain relatively low levels in most tissues (Figure 1B, adopted from [2]), except showing high expression in the brain suggesting an important role in brain development [3]. This finding is supported by the behavioral phenotype apparent in *ALK* gene knockout mice [4]. In mammals, ligands binding to the ALK protein are not well-defined, but pleiotropin (PTN) [5], Midkine (MK) [6] and FAM150A/B [7,8] are considered as potential interaction partners. Upon ligand binding, ALK receptors are dimerized, resulting in kinase domain phosphorylation and the activation of multiple downstream signaling pathways such as JAK/STAT3, PI3K/AKT, RAS/MAPK and PLC-γ pathways (Figure 1C).

The *ALK* gene has been found to be rearranged, mutated, or amplified in a series of tumors including anaplastic large cell lymphomas (ALCL), neuroblastoma, and non-small cell lung cancer (NSCLC) [9]. Chromosomal rearrangements are the most common alterations in this gene and result in creation of genetic fusions such as *EML4* (chromosome 2)-*ALK* (chromosome 2) [10], *RANBP2* (chromosome 2)-*ALK*, *ATIC* (chromosome 2)-*ALK*, *TFG* (chromosome 3)-*ALK*, *NPM1* (chromosome 5)-*ALK*, *SQSTM1* (chromosome 5)-*ALK*, *KIF5B* (chromosome 10)-*ALK*, *CLTC* (chromosome 17)-*ALK*, *TPM4* (chromosome 19)-*ALK*, and *MSN* (chromosome X)-*ALK* (Table 1). The incidence of ALK rearrangements is 3–7% in NSCLC [11,12], and novel rare ALK fusion partner genes (e.g., *GCC2*, *LMO7*, and *PHACTR1*) continue to be discovered [13]. Clinically and pathologically, the *ALK*-rearranged subtype of NSCLC forms a distinct entity among lung adenocarcinomas, usually characterized by young onset, non- or light-smoking history, poor-differentiation with acinar-predominant structure and with mucin/signet-ring cell pattern on histological analysis [14,15,16]. Characterization of the molecular subtypes of NSCLC revealed that *ALK*-rearranged NSCLC tends to be mutually exclusive with oncogenic *EGFR* and *KRAS* mutations [14]. Furthermore, unique microRNA expression signatures were found to distinguish *ALK*-rearranged NSCLC from *EGFR* and *KRAS* mutated NSCLC [17].

**Figure 2:**
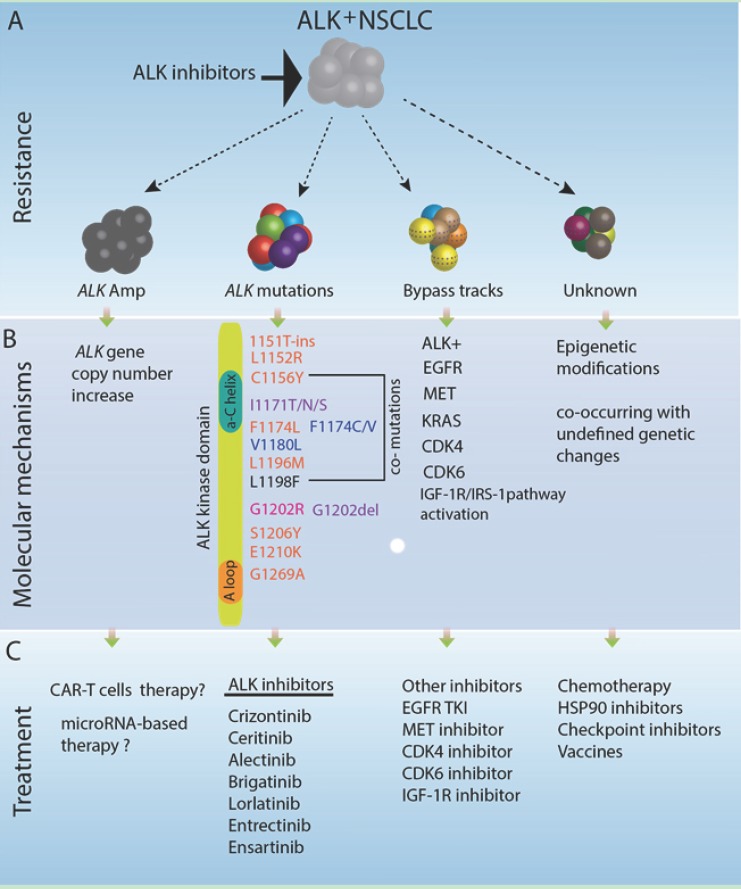
Resistance mechanisms in ALK fusion-positive lung cancer. A subset of lung cancer is driven by clonal *ALK*-rearranged genetic alterations. Top panel (**A**) depicts general categories of ALK tyrosine kinase inhibitor (TKI) resistance mechanisms. The oncogene-addicted ALK^+^ lung cancer cells initially respond to ALK tyrosine kinase inhibitors treatment (e.g., crizotinib treatment) followed by relapse due to acquired resistance, via *ALK* gene amplification, and/or *ALK* mutations, and/or bypass track activation or other unknown mechanisms. Middle panel (**B**) delineates the molecular mechanisms of each ALK TKI resistance phenotypes in addition to *ALK* rearrangement genetic alteration. Acquired mutations in ALK kinase domain are a well-known underlying molecular mechanism of ALK TKI resistance. Resistance mutations are listed here, highlighted with different colors: pink, crizotinib resistance; blue, alectinib resistance; and purple, ceritinib resistance. The ALK G1202R mutation is resistant to crizotinib, alectinib, and ceritinib. Rare compound mutations such as C1156Y and L1198F are resistant to loratinib, but sensitive to crizotinib, details see reference [19]. Bottom panel (**C**) lists treatment options for different types of ALK TKI resistance. Many FDA-approved ALK inhibitors (e.g., crizotinib, alectinib, and ceritinib) could be used to target acquired TKI resistance; other small molecules (e.g., EGFR inhibitors, MEK inhibitors, MET inhibitors, CDK4/CDK6 inhibitors or IGF-1R inhibitors) could be used to target bypass tracts with coexisting genetic alterations. Conventional chemotherapy, HSP90 inhibitor, immunotherapy and cancer vaccines are other options to treat the remaining subset of inhibitor-resistant ALK positive lung cancers. For *ALK* gene amplification resistant cases, chimeric antigen receptor-T cells therapy or microRNA-based therapy may be investigational treatment options.

The general features of ALK fusions that activate ALK function across the various fusion partners are: (1) the regulatory regions (e.g., active promoter) of the partner gene may initiate constitutive transcription of ALK fusion RNA resulting in overexpression of the ALK fusion protein; (2) unique domains in the partner proteins can influence the subcellular localization of ALK fusion proteins, which can reside in the nucleus, in the cytoplasm, and on cellular membranes; and (3) dimerization of ALK fusion proteins and thus activation of the ALK kinase domain through phosphorylation occurs in a ligand-independent fashion and is mediated by functional domains within the fusion partner (e.g., coiled-coil domains) or oligomerization at subcellular locations [18,19]. Since the discovery of the EML4-ALK fusion oncogene in lung cancer in 2007 [20,21], targeted therapies aiming to inhibit the constitutively activate ALK kinase domain have been the main focus for cancer therapy. The first small molecule ALK inhibitor, crizotinib, was approved by FDA in 2011. Second generation ALK inhibitors including ceritinib, alectinib, and brigatinib target both therapy-naïve and crizotinib-resistant ALK positive lung cancers with acquired ALK mutations, and next generation ALK inhibitors are in various stages of clinical trials [22]. The major challenge is that ALK^+^ NSCLCs initially respond to treatment but inevitably develop resistance to each ALK inhibitor, resulting in clinical relapse. The mechanisms of ALK inhibitor resistance are not completely understood, but *ALK* gene amplification, different mutations in the ALK kinase domain and bypass signaling pathways contribute to resistance (see recent review [22]) (Figure 2). Ongoing research in academia and the pharmaceutical industry aims to identify treatment options to overcome as well as delay or prevent resistance development, which is beyond the focus of this article. Here, we update the genomic landscape of ALK fusion-driven NSCLC, propose non-canonical ideas to manipulate the partner proteins in *ALK* fusions, and propose the design of novel immune-epitopes for potential ALK fusion targeted immunotherapy.

## 2. Genomic Characteristics of ALK Fusion-Driven NSCLC

ALK fusions in NSCLC are found in the majority of young, non- or light-smoking patients, and were originally reported to be mutually exclusive with KRAS or EGFR mutations [14,23]. With the application of next generation sequencing in the diagnosis of cancer patients, NSCLC cases harboring *ALK* genetic rearrangement concurrent with other somatic mutations have been detected. In contrast to previous reports, recent cases present co-occurrence of *ALK* fusion and *EGFR* mutations [24,25] as co-driver genetic alterations and indicate epistatic effects on malignant transformation or progression. Consistent with the notion of co-occurring genetic events in ALK^+^ lung cancer, Tan et al. reported [26] that 13 additional different somatic gene alterations coexist with ALK-fusion in 71 lung cancer cases analyzed. Recurrence of these gene mutations from high frequency to low frequency are *TP53*, *CDKN2A/B*, *SETD2*, *MYC*, *MCL1*, *RICTOR*, *EGFR*, *MSH6*, *MYST3*, *CCND1*, and *CDK4*. The vast majority of concurrent gene alterations are cell cycle related genes (*TP53*, *CDKN2A/B*, *MYC*, *CCND1*, and *CDK4*), followed by epigenetic modifying gene mutations (*SETD2* and *MYST3*) and survival and proliferative pathway genes (*MCL1*, *RICTOR*, and *EGFR*). Recent large genomic data analysis revealed 57 somatic co-mutations in NSCLC patients (*n* = 129) harboring EML4-ALK fusion protein; the top 10 co-mutated genes were: *TP53*, *ARID1A*, *ATPX*, *NF1*, *NOTCH1*, *ROS1*, *ARID1B*, *CSF1R*, *ALK*, *APC*, *FLT3*, *NPM1*, and *SMAD4* [13]. Of note, the aforementioned data were obtained from targeted DNA sequencing. Thus, it is anticipated that an additional order of magnitude of genetic aberrations co-occurring with ALK fusions in lung cancer could be detected with whole genome or whole exome sequencing analysis. These previously unappreciated co-occurring genetic alterations as well as the activation of IGF-1R-IRS-1 pathway [27] may cooperate with the oncogenic function of ALK in promoting lung cancer progression and therapy resistance in both the targeted therapy-naïve and the acquired resistance setting.

The genomic landscape of ALK rearrangements with other genetic alterations, though characterized in small cohorts to date, provides a rationale for potential combinatorial treatment of ALK inhibitors with other known or novel small molecule inhibitors (i.e., ALK/EGFR co-alterations, ALK/KRAS co-alterations or IGF-1R-IRS-1 pathway activation) [24,27,28]. It is also worth noting that epigenetic regulatory genes might be a promising therapeutic target in ALK fusion positive tumors. This is evident in recent studies [29], which demonstrated that specific lysine residues (e.g., 1451, 1455 and 1610) in EML4-ALK proteins were methylated by a lysine methyltransferase, SET and MYND domain-containing 2 (SMYD2). Preventing methylation of these lysine residues via either knockdown of SMYD2 or treatment with a SMYD2 inhibitor significantly attenuated the phosphorylation levels of EML4-ALK and inhibited downstream AKT activation. More strikingly, combination treatment with a SMYD2 inhibitor and an ALK inhibitor additively suppressed the growth of NSCLC cells, compared with single-agent treatment [29]. Therefore, the complexity of ALK fusion protein-mediated signaling, activated not only by constitutive phosphorylation within the kinase domain, but also by methylation of the ALK protein, together with epistatic effects of co-occurring mutations, could be of central importance for the development of potent next generation ALK inhibitors and the design of combinational therapeutic approaches.

In addition to co-alterations in coding genes in ALK^+^ lung cancer, studies have shown a unique signature of non-coding microRNA expression associated with ALK rearranged lung cancer [17,30,31]. Overexpression of *miR-1343-3p* and reduced expression of *miR-671-3p*, *miR-103a-3p*, *let-7e*, and *miR-342-3p* were especially distinctive in the *ALK*-rearranged lung cancer compared with *EGFR*-mutated and *KRAS*-mutated lung cancer [17]. Further, the ALK transcript represents a target of post-transcriptional, negative regulation by microRNAs. Using the miRWalk 2.0 algorithm (http://zmf.umm.uni-heidelberg.de/apps/zmf/mirwalk2/generetsys-self.html), 98 microRNAs were predicted to potentially bind to the ALK (Refseq ID: NM_004304) 3′ untranslated region (3′ UTR), which is present in the *ALK* fusion gene. If these microRNAs were experimentally verified, the small non-coding RNAs could be used to universally silence expression of wild type ALK or ALK fusion transcripts [31].

## 3. ALK Fusion Partner Proteins as Potential Therapeutic Targets

**Figure 3:**
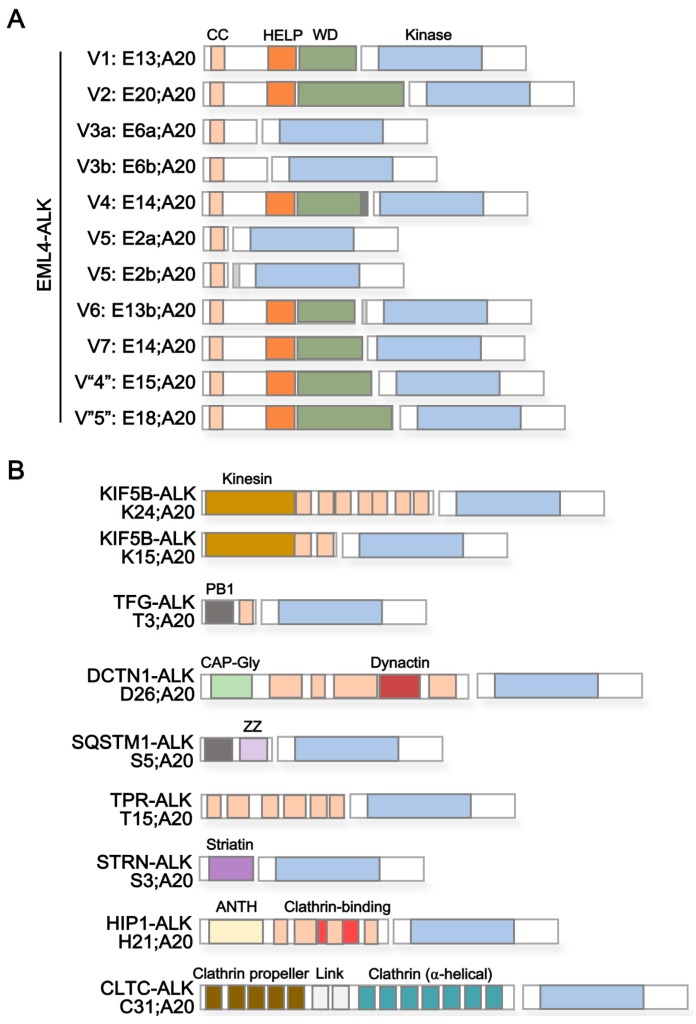
Schematic structural composition of ALK fusion proteins. (**A**) EML4-ALK fusion proteins. The breakpoint in the *ALK* gene is at exon 20; the breakpoint in EML4 is at different exons (e.g., 2, 6, 13, 14, 15, 18, and 20), resulting in variable EML4-ALK fusion proteins. In detail, variant 1 (E13;A20) and variant 2 (E20;A20) contain exons 1 to 13 and 1 to 20 of EML4, respectively, fused to exon 20 of ALK. Thus, the portion of EML4 includes its C-terminal coiled coil domain, HELP domain and parts of the WD repeat domain of EML4. Variant 3a/b EML4-ALK fusion proteins (E6a/b;A20) consist of exons 1 to 6 of EML4, with exon 6b being 188bp downstream of exon 6a, fused to exon 20 of ALK. Variant 4 (E14;E20) contain exons 1 to 14 of EML4, insertion of four amino acids of unknown origin, and fusion to exon 20 of ALK N-terminally lacking 49bp. Variant 5 EML4-ALK fusion proteins (E2a/b;A20) present a fusion of exon 2 of EML4 to exon 20 of ALK (E2a;A20) or to 117bp upstream of exon 20 of ALK (E2b;A20). Variant 6 (E13b;A20) contains exons 1 to 13 of EML4 fused to exon 20 of ALK with an insertion of 19 amino acids derived from upstream intron 19. Variant 7 (E14;A20) represents a genetic fusion of EML4 at exon 14 with ALK at exon 20, the latter lacking the first four amino acids. Variant “4” (E15;A20) is characterized as a fusion product of EML4 at exon 15 and ALK at exon 20. Variant “5” (E18;E20) consists of exons 1 to 18 of EML4 fused to exon 20 of ALK. (**B**) Other X-ALK fusion proteins. X represents any fusion partner including, amongst others, KIF5B-ALK (K24;A20 and K15;A20), TFG-ALK (T3;A20), DCTN1-ALK (D26;A20), SQSTM1-ALK (S5;A20), TPR-ALK (T15;A20), STRN-ALK (S3;A20), HIP1-ALK (H21;A20), and CLTC-ALK (C31;A20). For (**A**) and (**B**), protein domains are indicated by color and include: coiled coil (CC, light orange), HELP (dark orange), WD repeats (dark green), ALK kinase domain (blue), kinesin (ocher), PB1 (dark grey), CAP-Gly (light green), dynactin (dark red), zinc finger (ZZ, light violet), striatin (dark violet), ANTH (light yellow), clathrin-binding (light red), clathrin propeller (olive green), clathrin link (light grey), and clathrin (petrol) domains. Protein domains are derived from Pfam database [52]. See Table 1 for a list of ALK fusion partners and encoded protein domains.

Rearrangements of *ALK* with a variety of different cellular proteins have been identified (Table 1). Among the most abundant are fusions of the *ALK* gene with portions of the gene encoding the echinoderm microtubule-associated protein-like 4 (EML4) in lung cancer (Figure 3A) [20,32,33,34,35,36]. In addition, genetic fusions of *ALK* with genes such as *KIF5B*, *TFG*, *DCTN1*, *SQSTM1*, *TPR*, *CRIM1*, *STRN*, *HIP1*, *PTPN3*, *KLC1*, *CLTC*, and *FBXO36* have been discovered (Figure 3B) [21,22,26,33,37,38,39,40,41,42,43,44]. Besides differential capacity of ALK fusion variants regarding tumorigenesis in xenograft mouse models [45] as well as differential propensity to acquire additional mutations resulting in ALK TKI resistance (with more resistance-associated mutations in EML4-ALK variant 3 than non-variant 3) [46], the composition and characteristics of the ALK fusion partner can influence patient response to treatment with ALK inhibitors. This is exemplified in multiple reports indicating a better response of EML4-ALK variant 1 (E13;A20)- and variant 2 (E20;A20)-harboring patients compared to variant 3 (E6a/b;A20)-positive cases to ALK inhibitors, such as crizotinib, alectinib, or ceritinib [47,48,49,50]. Of note, Heuckmann et al. revealed that treatment response to ALK inhibitors correlates with protein stability of different EML4-ALK fusion proteins, with variant 3 (E6a/b;A20) as the shortest ALK fusion and with greater protein stability compared to other variants [47]. Analyzing the role of different protein domains present in EML4 regarding their effect on protein stability, the authors showed that deletion of the WD40 repeats but not the HELP domain resulted in a markedly increased stability of EML4-ALK variant 2 (E20;A20) fusions, mimicking the behavior of EML4-ALK variant 3 (E6a/b;A20) [47]. This indicates a significant negative role of the WD40 repeats on stability of the ALK fusion protein. Other studies have noted that the longer KIF5B-ALK fusion was markedly more sensitive than the shorter fusion, NPM-ALK, to ALK inhibitor treatment [47]. ALK fusion variants can also change protein structures (e.g., TAPE domain in the EML4 protein) of the partner proteins and require heat shock protein 90 to stabilize the resulting misfolded proteins. For example, EML4-ALK variant 1 and 2 contain a partial TAPE domain and become the substrate of HSP90; therefore, these ALK fusion proteins are sensitive to HSP90 inhibition, unlike EML4-ALK 3a/b and 5 [51]. This is further strengthened by synthetic lethality for combinatorial HSP90 inhibition upon treatment with the ALK inhibitor crizotinib, opening the possibility to overcome de novo treatment resistance of ALK fusion proteins that show an increased stability under crizotinib treatment alone [47].

Targeting proteins interacting with EML4-ALK might improve treatment response to ALK inhibitors such as crizotinib or alectinib. In a recent study by Zhang et al., an EML4-ALK interactome was defined and additional proteins that mediate an increased response to ALK inhibitor upon knockdown have been identified [53]. The latter set of genes comprises scaffolding proteins, such as FRS2 (Fibroblast growth factor receptor substrate 2) and CC2D1A (Coiled-Coil and C2 Domain Containing 1A) [53]. These scaffolding proteins may participate in ALK-fusion protein-mediated downstream pathway activation; therefore, combinatorial inhibition of the interaction of EML4-ALK and the respective signaling protein(s) by competitive binding peptides or inhibitors as well as by downregulation/degradation of scaffolding proteins might improve therapeutic response of EML4-ALK driven NSCLC patients to ALK inhibitor therapy.

Besides an important role of functional domains contained in the fusion partner for treatment response to ALK inhibitors, their composition is equally important to ALK-mediated oncogenesis. As outlined in Table 1, the majority of ALK fusion partners contain coiled-coil domains—central mediators of the oligomerization of proteins and often found in cytoskeletal proteins and associated motor proteins [54]. The fusion of proteins harboring a coiled-coil-domain with the kinase-encoding portion of ALK most likely allows dimerization and constitutive activation of ALK, as previously noted [21]. This was confirmed by Soda et al. when studying the functional relevance of EML4 protein domains present in the EML4-ALK variant 1 fusion protein (E13;A20) regarding the transforming ability of the ALK fusion protein [20]. EML4-ALK variant 1 expression constructs encoding the full length fusion protein or fusion proteins lacking the basic domain (Δ31–140 including deletion of coiled-coil domain at residues 17–51), the HELP domain (Δ220–296), or the WD40 repeats (Δ305–475) were transfected into 3T3 cells and their ability to form tumors was assessed in vivo [20]. While deletion of WD40 repeats did not impair tumor formation, deletion of the EML4 HELP domain resulted in reduced tumor outgrowth [20]. Most importantly, deletion of the basic domain, generating an EML4 protein lacking its N-terminal coiled-coil domain, completely abolished tumor formation in vivo—again confirming the central role of protein dimerization via the coiled-coil domain of the ALK fusion partner in constitutive ALK signaling and ALK-mediated oncogenesis [20]. Further, the reduction of tumor outgrowth upon deletion of the HELP domain may point to an additive role of the EML4 fusion partner in driving oncogenesis. Recent evidence published by our laboratory indicated that EML4-ALK fusion proteins engage Ras-MAPK signaling via the EML4 HELP domain [55]. In particular, deletion of the HELP domain contained within EML4 in the EML4-ALK variant 1 (E13;A20) fusion protein impaired the intracellular localization of the fusion protein and diminished GTP loading of Ras, resulting in reduced Ras-MAPK signaling [55]. Of note, cases positive for EML4-ALK fusion proteins that contain the EML4 HELP domain might show an improved response to rational combinational therapy targeting MAPK signaling in addition to ALK directly, as outlined in our recent publication [55].

In addition to EML4, gene ontology (GO) enrichment analysis confirms an over-representation of microtubule or microtubule-associated proteins among ALK fusion partners (data not shown). Oligomerization and activation of ALK due to distinct intracellular localization at microtubules (EML4, KIF5B, TFG, DCTN1, PTPN3, and KLC1) or cellular membranes (SQSTM1, TPR, CRIM1, STRN, HIP1, and CLTC) is likely to be of central relevance for ALK-mediated oncogenesis and presents a new therapeutic target for the treatment of ALK fusion driven cancers. Pharmacologic compounds that impair the formation of ALK signaling complexes need to be developed and tested to evaluate this hypothesis directly. Amongst others, microtubule-destabilizing agents are already approved as single or combination therapy in solid and hematologic cancers [56], but their discrete benefit in ALK fusion positive lung cancer has not been studied. Similarly, the inhibition of kinesin and dynactin domain-mediated association of ALK fusion proteins with microtubules might be beneficial for KIF5B-DCTN1-or KLC1-ALK harboring cases, with inhibitors already being available but not tested in ALK^+^ NSCLC [57,58,59,60]. Most importantly, novel coiled coil mimetics or other helical peptides and peptidomimetic inhibitors that hamper oligomerization of ALK fusion associated signaling proteins offer another promising approach [61,62]. As shown for Bcl-Abl proteins, targeting protein dimerization by interfering with the coiled-coil domain significantly impacts fusion protein-driven oncogenesis [63]. In the context of the relevance of protein dimerization for ALK-mediated signaling and oncogenesis of ALK fusion protein, the use and therapeutic benefit of similar drugs as single-agent or combinational therapy in ALK fusion positive NSCLC warrants evaluation.

## 4. Immunotherapy for ALK^+^ Lung Cancer

Immunotherapy aims to establish or enhance an effective immune response against tumor cells. This could be accomplished via different strategies, including tumor vaccination, adoptive transfer of immune cells, and modification of the immune system to boost an established immune response [64]. The conventional immunotherapy such as vaccination against tumor-associated antigens exhibited limited anti-tumor effect in the past [65]. With the discovery of inhibitory pathways in immune cells, so called checkpoint molecules, and the development of antibody-based blockades against these checkpoint molecules such as cytotoxic T lymphocyte-associated protein 4 (CTLA-4) and programmed cell death protein (PD-1)/PD1 ligand 1(PD-L1), cancer immunotherapy is entering a new era [66]. Recently, immune checkpoint inhibitors (ICI) against PD-1 have been tested in NSCLC, and an objective response rate of up to 20% has been observed in selected patients with high PD-L1 expression [67,68,69]. In general, patients with squamous NSCLC respond to ICI better than patients with non-squamous-NSCLC, and, amongst the latter, a better response for lung adenocarcinoma patients with a heavy smoking history than those with light- or non-smoking record has been reported [70]. The differences between “smoker” and “non-smoker” NSCLC patient cohorts that are causally linked to their ICI response may include the following: (1) genomically, smokers usually harbor higher mutational burden and thus have a higher number of expressed neoantigens than non-smokers; and (2) the tumor microenvironment (TME) in smokers is characterized by an increased frequency of active, tumor-infiltrating CD8^+^ T cells as well as increased levels of anti-tumor cytokines such as IFN-γ and granzyme, while the TME in non-smokers shows elevated immunosuppressive features such as a high number of FOXP3^+^ regulatory T cells, the accumulation of M2-like macrophages and less activated effector CD8^+^ T cells [71]. ALK rearranged lung adenocarcinoma is characterized by patients with non- or light-smoking history and mutational burden in this subtype of NSCLC is incompletely characterized; intuitively, it is inferred that the number of non-synonymous mutations is lower than median number of 192 in lung cancers more generally [72]. In current clinical trials of lung cancer immune checkpoint blockade treatment, patients with ALK fusion and EGFR mutation have shown minimal responses [67,69]. Experimental evidence showed that lung cancers with ALK fusion harbor an immune-suppressive TME and a T-cell exhausted state [73]. Therefore, converting the TME in ALK^+^ NSCLC to such extent that an effective immune response against the tumor will become possible remains an important challenge.

How might the immune-unresponsive TME be converted to a responsive TME? Neoantigen-based cancer vaccination could activate T cells and promote tumor-associated infiltrating effector lymphocytes into the TME [74].

Tumor neoantigens are the products of all genetic alterations accumulated in cancer genome during tumorigenesis. The neoantigens are superior to other tumor-associated antigens due to: (1) neoantigens are presented only in tumor cells; (2) neoantigens are not subject to central tolerance in the thymus; and (3) neoantigens produce strong immunogenicity, with persistent cytotoxic T cell activation. These antigens can arise not only from missense mutations but also fusion transcripts [75] or any altered open reading frame that encodes novel stretches of amino acids that are not encoded in the normal genome.

As we mentioned in the introduction section, ALK expression is normally restricted to a subset of cells in the nervous system. Its pathological expression in lung cancer and most other malignancies is due to chromosomal translocations that lead to the formation of an ALK-derived oncogenic fusion proteins, which are overexpressed and constitutively activated in cancer cells. The high level of ALK fusion protein expression in cancer cells and its direct role in tumorigenesis, in line with the fact that normal ALK is expressed at low levels in the immune privileged nervous system, makes it an ideal tumor-specific target for immunotherapy in ALK fusion-positive NSCLC. The presence of anti-NPM-ALK antibodies in ALCL patients has been reported and indicates that patients are able to respond to ALK-derived neoantigens [76]. From the ALK kinase domain, two HLA-A2.1 restricted CD8^+^ T-cell epitopes, p280–289 (SLAMLDLLHV) and p375–389 (GVLLWEIFSL) were identified and confirmed to be immunogenic in HLA-matched ALCL and neuroblastoma cell lines [77]. In principle, these peptides could be used for vaccinations against ALK fusion antigens in cancer patients including NSCLC; however, more experimental tests on humanized mouse or clinical trials need to be performed. Chiarle et al. [78] have shown that DNA vaccination with plasmids encoding portions of the cytoplasmic domain of ALK, which has been translocated in different fusion proteins that are necessary for the growth of ALCL, protects mice from local and systemic lymphoma growth. The protection is potent and durable by eliciting an ALK-specific interferon-gamma response and CD8^+^ T cell-mediated cytotoxicity. A combination of chemotherapy and vaccination significantly enhanced the survival of mice challenged with ALK^+^ lymphomas [78]. These findings indicate that ALK represents a compelling tumor antigen for vaccination-based therapies of ALCL and possibly other ALK^+^ human tumors. The efficacy of DNA-based vaccination was also observed in lung cancer xenograft models with EML4-ALK as well as in a TFG-ALK transgenic lung cancer mouse model [73].

In animals with high tumor burden, the anti-tumor effect of an ALK vaccine is diminished. This may correlate with an exhausted phenotype of T cells and PD-L1 expression. Thus, the combination of an ALK vaccine and ALK inhibitors, as expected, increased the anti-tumor efficacy [73] due to an increase of neoantigens and possible tumor microenvironment changes (e.g., more tumor-infiltrating lymphocytes). PD-L1 expression is increased in ALK^+^ lung cancer cells through downstream pathway signaling, in particular ERK and AKT activation but not JAK/STAT3/5 signaling [79]. Given a higher objective response rate of anti-PD-1 and anti-CTLA-4 in PD-L1 positive lung cancer, it is reasonable to consider adding anti-PD-1 and/or anti-PD-L1 antibodies to the treatment of ALK^+^ lung cancer, particularly at the time of resistance to ALK inhibitor monotherapy.

When we analyzed the ALK fusion with various partners listed in Figure 3 and Table 1, we noted that some gene rearrangements include extra sequence from introns or insertions (e.g., EML4-ALK variant 3a/b, 4, and 5b); such DNA alterations create new protein peptides that might be immunogenic. In addition, there are more than 20 different mutations in ALK fusion proteins [80]; whether these mutated peptides could be developed into therapeutic vaccines remains to be investigated.

Therapy with autologous T cells that have been genetically-engineered to express chimeric antigen receptors (CAR) or T cell receptors (TCR) provides a feasible and emerging treatment for certain cancer patients [81]. Since amplification of ALK (native) can result in ALK TKI resistance, an antibody against extracellular domain of ALK receptor is alterative potential approach to treatment [82,83]. Moreover, a chimeric antigen receptor of T cells targeting the ALK protein could provide a novel adoptive T-cell-targeted therapy. For example, T cells expressing a CAR incorporating a scFv of the ALK48 mAb linked to a 4-1BB-CD3ζ signaling domain elicited ALK-expressing tumor lysis and produced IFN-γ upon antigen stimulation, but limited anti-tumor efficacy was observed in xenograft models of human neuroblastoma [84].

Taken together, *ALK* rearranged lung cancer appears to exhibit an immune-suppressive or anergic tumor microenvironment. Neoantigens produced from ALK fusion proteins or other co-occurring gene mutations may stimulate the host immune system and re-reactivate effector T cells. Neoantigen-based vaccines could provide an alternative therapeutic option to treat refractory ALK^+^ cancers such as lung cancer.

## 5. Conclusions and Future Perspectives

*ALK* gene rearrangements are present in 3–7% of NSCLC. The fusion proteins encoded by *ALK* fusion genes are constitutively activate kinases and drive various oncogenic functions [13,45]. Since the identification of first EML4-ALK aberrations in lung cancer in 2007 [20], ALK-specific inhibitors (e.g., crizotinib) have been developed. Lung cancer patients harboring ALK fusion initially respond to TKIs, leading to tumor regression, but tumors inevitably relapse due to multiple resistance mechanisms such as acquired mutations in ALK, ALK amplification, and the aforementioned concurrent genetic alterations within the cancer genome. Interestingly, the composition of ALK fusion proteins determines sensitivity to ALK inhibitor treatment and patient outcome during therapy. Fusion partners of ALK play a central role in mediating dimerization and constitutive ALK signaling, both by facilitating distinct intracellular localization(s) as well as protein oligomerization. Further, ALK fusion partners themselves might engage additive signaling pathways that support ALK-mediated oncogenesis.

While there is a strong focus on the development of next generation inhibitors targeting emerging resistance mutations, we propose a non-canonical strategy to fight ALK fusion positive NSCLC. In brief, we propose: (1) to target specific domains in partners of ALK fusion proteins to inactivate ALK kinase function; (2) to trigger a specific immune response against ALK-fusion proteins (e.g., via peptide vaccines); and (3) to develop combinatorial treatment approaches that can target coexisting genetic alterations in ALK positive NSCLC. Although these hypotheses need to be tested through multiple avenues before moving to the clinic, we are optimistic that these ideas may have clinical implications by expanding the therapeutic options for lung cancer patients.

## Figures and Tables

**Table 1:** Characterization of ALK fusion partners in NSCLC.

Protein Name ^1^	Reported ALK Fusion		Uniprot ID	Molecular Function ^2^	Subcellular Localization ^3^	Protein Domains ^4^
Echinoderm microtubule-associated protein-like 4 (EML4)	E2a/b;A20	[36]	Q9HC35	Microtubule binding and assembly	Cytoplasm > cytoskeleton	Coiled coil [aa17–51] HELP [aa225–297] WD40 repeats [aa229–347, 499–537, 583–620, 711–749, 824–863]
	E6a/b;A20	[32]				
	E13;A20	[20]				
	E13b;A20	[33]				
	E14;A20	[33,36]				
	E15;A20	[34]				
	E18;A20	[35]				
	E20;A20	[20]				
Kinesin-1 heavy chain (KIF5B)	K24;A20 K15;A20	[33] [43]	P33176	Microtubule-associated motor protein	Cytoplasm > cytoskeleton	Kinesin [aa14–325] Coiled coil [aa330–364, 418–543, 594–684, 691–711, 716–757, 768–802, 825–912]
TRK-fused gene (TFG)	T3;A20	[21]	Q92734	Dynamic interaction of endoplasmic reticulum and microtubules	Endoplasmic reticulum (ER)	PB1 [aa10–93] Coiled coil [aa102–122]
Dynactin subunit 1 (DCTN1)	D26;A20	[37]	Q14203	Dynein-mediated retrograde transport of vesicles and organelles along microtubules	Cytoplasm > cytoskeleton > microtubule	CAP-Gly [aa29–94] Coiled coil [aa217–352, 360–383,388–540, 952–1043, 1185–1205] Dynactin [aa527–805]
Sequestosome-1 (SQSTM1)	S5;A20	[37]	Q13501	Autophagy receptor, endosome organization	Cytoplasm > late endosome, autolysosome/-phagosome	PB1 [aa21–102] Zinc finger [aa122–165] UBA [aa379–440]
Nucleoprotein TPR	T15;A20	[38]	P12270	Scaffolding element in nuclear pore complex, nucleocytoplasmic transport	Nucleus > Nuclear pore complex	Coiled coil [aa29–49, 61–172, 219–281, 293–366, 427–513, 543–570, 576–596, 665–752, 760–801, 827–868, 887–914, 934–982, 997–1071, 1096–1116, 1149–1169, 1221–1238, 1269–1303, 1311–1345, 1354–1416, 1469–1552, 1564–1598, 1603–1630] TPR/MLP1/MLP2 [aa1038–1165]
Cysteine-rich motor neuron 1 protein (CRIM1)	n/a	[26]	Q9NZV1	Tissue development, interaction with transforming growth factor beta family proteins	Cell membrane	Single pass type I transmembrane [aa1–34] IGFBP [aa37–90] VWC [aa336–390, 403–456, 608–662, 679–734, 753–808, 819–873] Antistatin [aa469–498, 505–532, 539–564, 567–592]
Striatin (STRN)	S3;A20	[39]	O43815	Calmodulin-binding protein involved in scaffolding and signaling	Cytoplasm, Cell membrane	Striatin [aa48–177] Coiled coil [aa67–115] WD40 repeats [aa453–491, 506–544, 559–597, 696–734, 738–779]
Huntingtin-interacting protein 1 (HIP1)	H21;A20	[40]	O00291	Involved in clathrin-mediated endocytosis and trafficking	Cytoplasm, Endomembrane system, nucleus	ANTH [aa39–308] Coiled coil [aa377–397, 409–488, 496–555, 584–611, 978–1007] Clathrin binding [aa482–580] I/LWEQ [aa862–1010]
Tyrosine-protein phosphatase non-receptor type 3 (PTPN3)	Chimeric fusion	[44]	P26045	Tyrosine phosphatase, interaction with cytoskeleton	Cell membrane, cytoplasm > cytoskeleton	FERM [aa33–97, 113–222, 226–316] PDZ [aa510–595] Y phosphatase [aa670–900]
Kinesin light chain 1 (KLC1)	K9;A20	[41]	Q07866	Microtubule-associated motor protein	Cytoplasm > cytoskeleton	n/a
Clathrin heavy chain 1 (CLTC)	C31;A20	[22]	Q00610	Intracellular trafficking and endocytosis	Cell membrane, cytoplasm > cytoskeleton	Clathrin propeller repeat [aa19–56, 148–187, 198–234, 253–288, 296–330] Clathrin (H) link [aa331–354, 356–421] Clathrin [aa537–679, 686–827,835–971, 979–1123, 1128–1268, 1274–1419, 1424–1565]
F-box only protein 36 (FBXO36)	n/a	[42]	Q8NEA4	Substrate recognition in E3 ubiquitin ligase complex	n/a	F-box [aa92–139] Coiled coil [aa166–186]

^1,2,3^ Information retrieved from UniProt database (http://www.uniprot.org), accessed on 9 April 2017 [85]; ^4^ Information retrieved from Pfam database (http://pfam.xfam.org), accessed on 9 April 2017 [52]. Protein domains are underlined and include coiled-coiled domain, hydrophobic EMAP-like protein (HELP) motif, WD40 repeats, kinesin domain, PB1 domain, cytoskeleton-associated protein (CAP)-Gly domain, dynactin motif, ZZ-type zinc finger domain, ubiquitin associated (UBA) domain, TPR/myosin-like protein (MLP) 1/2 motif, single pass type I transmembrane domain, insulin-like growth factor binding protein (IGFBP) motif, von Willebrand factor type C (VWC) domain, antistatin domain, striatin domain, ANTH domain, clathrin binding domain, I/LWEQ domain, FERM domain (F, 4.1 protein; E, ezrin, R, radixin; M, moesin), PDZ domain, Y phosphatase domain, clathrin propeller repeat, clathrin (H) link, clathrin domain, and F-box domain.

## Abbreviations

The following abbreviations are used in this manuscript:

ALCL	Anaplastic Large Cell Lymphoma
ALK	Anaplastic Lymphoma Kinase
CAR-T	Chimeric Antigen Receptor-T cell
NSCLC	Non-Small Cell Lung Cancer
TKI	Tyrosine Kinase Inhibitor
